# Functional outcome of modified Pauwels’ intertrochanteric osteotomy and total hip arthroplasty in femoral neck fractures in elderly patients

**DOI:** 10.4103/0019-5413.38581

**Published:** 2008

**Authors:** Narender K Magu, Rochak Tater, Rajesh Rohilla, Ashish Gulia, Roop Singh, Pardeep Kamboj

**Affiliations:** Department of Orthopedics, Pt. B. D. Sharma PGIMS, Rohtak, Haryana, India

**Keywords:** Subcapital femoral neck fracture, femoral neck fracture in elderly, osteoporosis, modified Pauwels’ intertrochanteric osteotomy, total hip arthroplasty

## Abstract

**Background:**

A high union rate (75%-100%) with a lower incidence of avascular necrosis (8%-9.3%) has been reported with intertrochanteric osteotomy in femoral neck fractures in elderly whereas arthroplasty eliminates the incidence of nonunion and avascular necrosis We present a series of femoral neck fracture in elderly treated with modified Pauwels’ intertrochanteric osteotomy and total hip arthroplasty for their functional outcome.

**Materials and Methods:**

29 elderly patients of 60 years and above sustaining fresh subcapital femoral neck fracture underwent total hip arthroplasty (group I, n=14) and modified Pauwels’ intertrochanteric osteotomy (group II, n=15). Functions were evaluated using modified Harris hip score, d'Aubigne and postel criteria and SF-36 score at 6, 12, 52 and 100 weeks.

**Results:**

The fracture union in group II was achieved in 14 (93.3%) patients at the fracture site at an average of 15 weeks and osteotomy united in all patients. Avascular necrosis of the femoral head was observed in one patient (6.7%). Average operative time was 88.9 and 65.6 minutes in group I and II, respectively (P value = 0.00001). An average of 0.8 and 0.2 unit blood was transfused in patients in group I and II, respectively (P value = 0.001). Average time of full weight bearing was 6.1 weeks and 11.6 weeks in group I and group II, respectively. At 100 weeks 71.4% (n = 10) patients in group I and 80% (n = 12) patients in group II showed good to excellent results on the basis of modified Harris hip score. 71.4% (n = 10) patients in group I and 66.6% (n = 10) patients in group II showed good to excellent results on the basis of d'Aubigne criteria. Average SF-36 score was 17.2% in group I and 17.6% in group II. Revision osteotomy was performed in one patient in group II because of implant cut through. Another patient in group II underwent THR because of painful hip. One patient in group I presented with dislocation after 3 weeks of surgery.

**Conclusion:**

Functional results of total hip arthroplasty and intertrochanteric osteotomy are comparable and the valgus intertrochanteric osteotomy with osteosynthesis in subcapital femoral neck fractures in elderly patients of sixty years and above may be considered as an option.

## INTRODUCTION

Various methods of internal fixation for the management of subcapital femoral neck fracture are reported with fewer postoperative complications, a shorter hospital stay and reduced cost of treatment.^1^–^7^ However adequate fixation is not always achieved particularly in patients with osteoporosis.^8^ Higher rate of failed osteosynthesis (21%), nonunion (20-35%) and avascular necrosis (10-17%) subjecting the patient for revision surgery in 20%-36% has been reported.^9^,^10^ Arthroplasty allows early mobilization, weight bearing and eliminates nonunion and avascular necrosis.^11^ However stem loosening (11-12%), acetabular erosion (4-20%), acetabular protrusion (7-11%), mortality (15-23%), infection (0-18%), dislocation (6-18%) and revision surgery (6.75-24%) are known complications of arthroplasty.^8^,^10^,^12^,^13^ The results of osteosynthesis and arthroplasty are also found to be comparable.^14^ However, success rates following internal fixation of displaced subcapital femoral neck fractures are lower than for undisplaced ones.^15^,^16^ A high union rate (75%-100%) with a lower incidence of avascular necrosis (8%-9.3%) has been reported by various authors with intertrochanteric osteotomy.^17^–^19^ No comparative study of abduction osteotomy with total hip arthroplasty has been reported. We have performed modified Pauwels’ intertrochanteric osteotomy and total hip arthroplasty in femoral neck fractures in elderly patients and the study reports the functional outcome of both group of patients.

## MATERIALS AND METHODS

29 consecutive patients of fresh femoral neck fracture of sixty years or above were included in this retrospective analysis. The patients of intracapsular femoral neck fracture with Garden^20^ stage 3 and 4 or Pauwels^21^ type 2 and 3 constituted the clinical material. The patients were advised total hip replacement (THR). Those patients who underwent THR were included in group I (n = 14) while in rest modified Pauwels’ intertrochanteric osteotomy was done and included in group II (n = 15). In group I two patients had rheumatoid arthritis and one patient had osteoarthritis of both knees. Plain antero-posterior radiograph of pelvis including both hips in 15 degree internal rotation was taken to study Singh's index^22^ from normal hip. Patients were operated after taking a formal consent and preanaesthetic check up. Pre-operative planning was done to define Pauwels’ angle and to select an appropriate implant in osteotomy group. The group I (THR group) patients were operated with modified Hardinge approach.^23^

**Figure 1:**
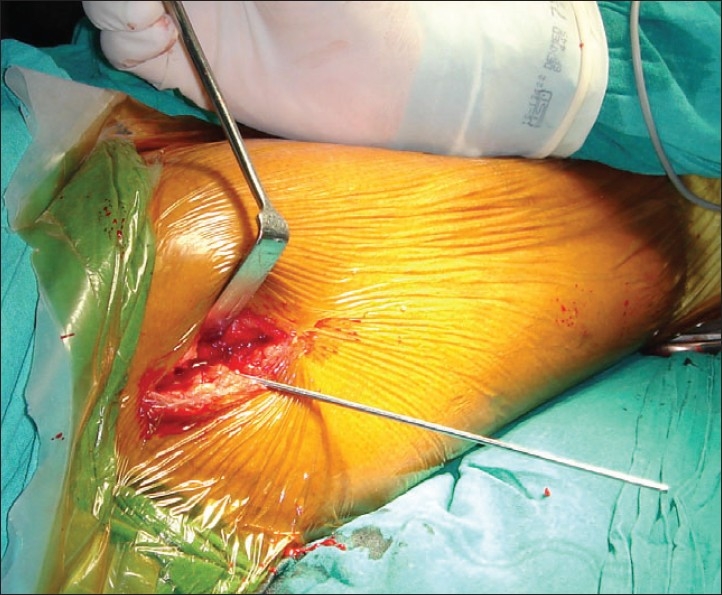
Surgical technique of modified Pauwels’ intertrochanteric osteotomy. Shows initial two inches long incision and guide wire insertion

**Figure 2:**
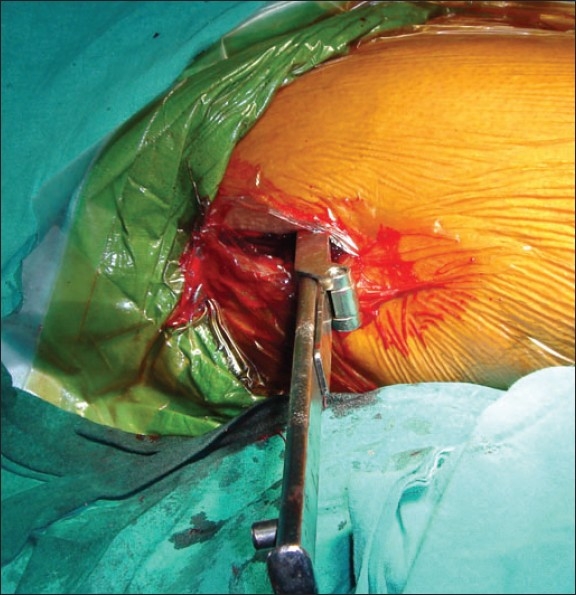
Shows insertion of the AO chisel into the postero-inferior quadrant of the head of the femur after stabilization of the fracture with a 6.5 mm cancellous lag screw

**Figure 3:**
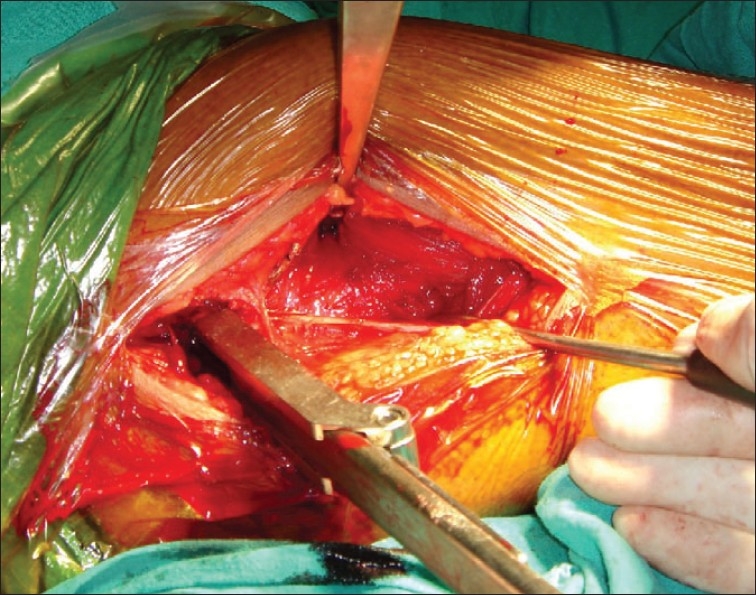
Shows extension of the incision for osteosynthesis of the osteotomy with double angle blade plate

**Figure 4:**
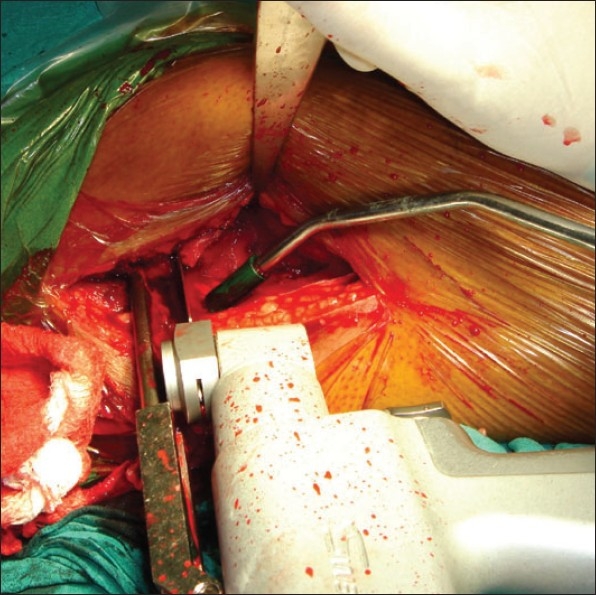
Shows intertrochanteric osteotomy being performed with an oscillating saw

**Figure 5:**
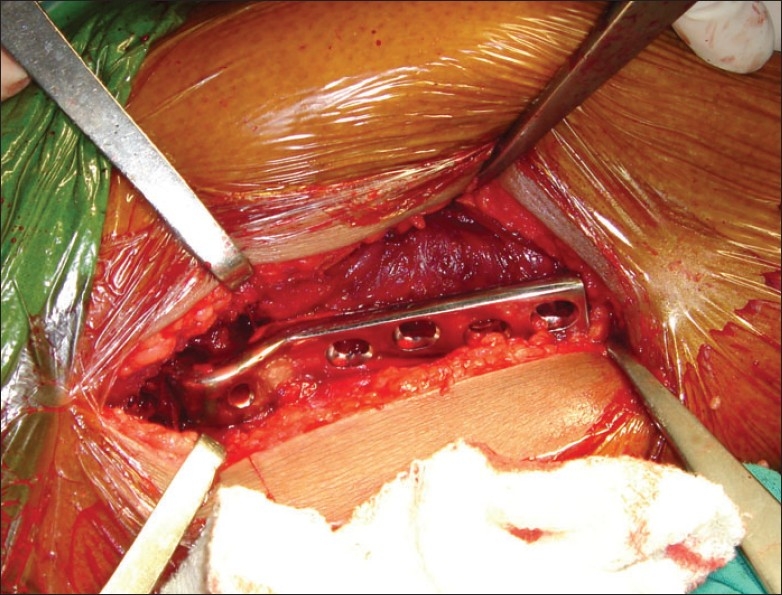
Double angle osteotomy blade plate in position before insertion of the screws in the shaft of the femur to complete the procedure

In group II patients the fracture was reduced by traction and internal rotation under image intensifier on a conventional operation table. A longitudinal skin incision approximately 2 inches in length starting from the tip of the greater trochanter was made [Figure 1]. First guide wire was passed into the inferior and posterior quadrant of the femoral head. Second guide wire was passed parallel and proximal to the first guide wire to maintain the reduction at the fracture site. Fracture was stabilized with an appropriate length 16 mm threaded 6.5 mm cancellous lag screw. Entry site for AO chisel was made with an osteotome at the trochanter tubercle. AO chisel was inserted into the inferior and posterior quadrant of head of femur [Figure 2]. The skin incision was enlarged [Figure 3] and proximal osteotomy was done parallel to the AO chisel using oscillating saw [Figure 4]. Distal osteotomy was done directing the saw medially, upwards and obliquely above the lesser trochanter to excise an intertrochanteric wedge of 20°-30° with the AO chisel still *in situ*. The blade of the double angled osteotomy blade plate was inserted in femoral head up to subchondral area along the track made with the AO chisel. Osteotomy was closed by abducting the limb and osteosynthesis of femoral neck fracture and osteotomy was achieved [Figure 5]. Wound was closed in layers over a negative suction drain. Time taken for surgery and number of blood transfusions were recorded for the patients in both the groups. The patients were encouraged to do quadriceps exercises, non weight bearing hip mobilization after surgery and were permitted partial weight bearing after 6 weeks and full weight bearing after 12 weeks. Patients in group I were made to stand with support and encouraged to bear weight on the affected limb on 5^th^ post operative day and were discharged on 12^th^ postoperative day. Functions were evaluated using modified Harris hip score,^24^ d'Aubigne and Postel^25^ criteria and SF-36 score^26^ at 6, 12, 52 and 100 weeks.

Data were analyzed with Chi-square test, Fisher's exact test and independent t test. For all tests, probability less than 0.05 were considered significant.

## RESULTS

In group I average age of the patients was 66.5 years (60-85 years) whereas in group II it was 67.4 years (60-82 years) (*P* value = 0.76) [Table 1]. In group I both sides were equally involved whereas in group II left side was involved in 53.3% (n = 8) patients and right in 47.7% (n = 7) (*P* value = 0.85) [Table 2]. All patients sustained fracture due to trivial trauma except for two patients in group II who sustained injury due to moderate trauma. Severe degree of osteoporosis (Singh's index grade I, II, III) was seen in all the patients except for one patient in group I (*P* value = 0.17). All the patients in both groups were of Garden's stage IV except one patient of type III in group I (*P* value = 0.29). 42.9% (n = 6) patients in group I and 60% (n = 9) patients in group II were of Pauwels’ type III and remaining were of Pauwels’ type II (*P* value = 0.35). Average time interval between injury and operation was 13 days in group I where as it was 14.3 days in group II (*P* value = 0.64). In group II an average of 23.3^°^ (20^°^-30^°^) intertrochanteric wedge was excised and an average neck shaft angle of 138.6^°^ (128^°^-160^°^) was achieved. Post operatively femoral head was anteverted in 46.7% (n = 7) patients, retroverted in 33.3% (n = 5) patients and neutral in 20% (n = 3) patients in group II. Average operative time was 88.9 and 65.6 minutes in group I and II respectively (*P* value = 0.00001, significant). Blood transfusion was required in 11 patients in group I where as in group II patients it was required only in 3 patients. An average of 0.8 and 0.2 unit blood was transfused in patients in group I and II respectively (*P* value = 0.001, significant). All the patients in group I were discharged only after stitch removal. In group II 14 out of 15 patients were discharged after 48-72 hours. In group I the average time period for partial weight bearing was 10.7 days and in group II it was 44.5 days post surgery (*P* value = 0.000002). Full weight bearing was started in an average time period of 6.1 weeks and 11.6 weeks by group I and group II patients respectively (*P* value = 0.0000003). In group II union was achieved in 14 out 15 patients (93.3%) at the fracture site at an average of 15 weeks while union was achieved in all the patients at osteotomy site at an average of 14 weeks [Figure 6A–B]. Revision osteotomy was performed in one patient in group II because implant was cut out from femoral head. This was probably due to an unacceptable position of the tip of the blade in the femoral head. Another patient in group II underwent THR because of painful hip resulting from implant into the joint, non union and avascular necrosis of femoral head. One patient in group I presented with dislocation after 3 weeks of surgery [Figure 7a–c]. In group I one patient showed shortening of 1 cm and another patient showed lengthening of 1 cm. In group II one patient showed shortening of 2 cm and another patient showed lengthening of 1 cm. 11 out of 15 patients (73.3%) in group II showed external rotation deformity. Average external rotation deformity was 10.3^°^ (10^°^-20^°^). One patient in group II developed flexion contracture of 35^°^. There was no incidence of mortality or infection in the series at the end of the follow up of 2 years.

**Figure 6A:**
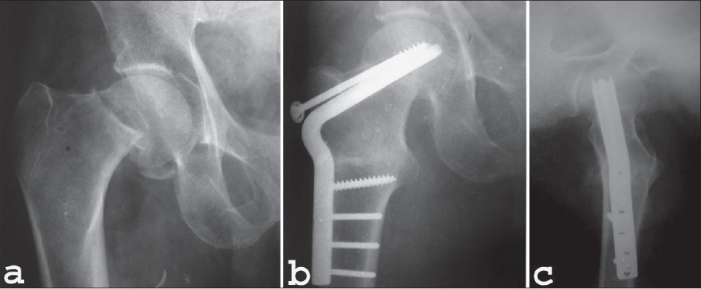
A: Antero-posterior radiograph (a) of 80 years male shows Garden stage IV and Pauwels type III femoral neck fracture. Anteroposterior radiograph (b) of the same patient after 2 years of modified Pauwels’ intertrochanteric osteotomy, shows sound union of fracture and of osteotomy. Femoral head is viable. Frog leg radiograph (c) of the patient at the 2 years of follow up

**Figure 6B:**
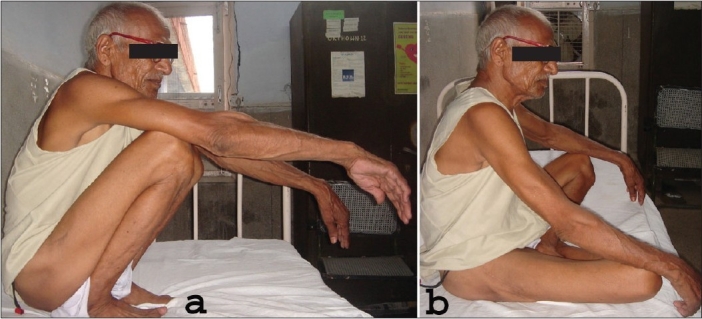
Clinical photograph of the patient squatting on toes (a) and sitting cross leg (b)

**Figure 7:**
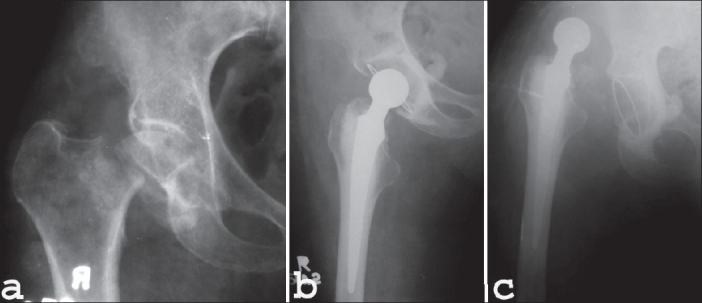
Antero-posterior radiograph (a) of 60 years female with Garden stage IV and Pauwels type II subcapital femoral neck fracture. Immediate postoperative antero-posterior radiograph (b) of the same patient after total hip arthroplasty. Antero-posterior radiograph (c) at 3 weeks showing dislocation of the prosthesis. The acetabular component tilted vertically from its initial position. Poor cementing of the acetabular component resulted in dislocation of the hip

**Table 1:** Comparative values in the two groups

	Group I Mean ± S.D.	Group II Mean ± S.D.	*P* value	t value
Age (years)	66.5 ± 6.7	67.4 ± 7.8	0.76	−0.30
Time interval between injury and operation (days)	13 ± 7.18	14.3 ± 7.16	0.64	−0.47
Operative time (minutes)	88.9 ± 10.1	65.6 ± 12.9	0.00001	5.41
Blood transfusion (units)	0.8 ± 0.5	0.2 ± 0.4	0.001	3.68
Partial weight bearing (days)	10.7 ± 7.0	44.5 ± 18.2	0.000002	−6.66
Full weight bearing (weeks)	6.1 ± 2.3	11.6 ± 2.0	0.0000003	−6.73
Harris hip score at 100 weeks	86 ± 17.9	85.5 ± 20.0	0.94	0.06
d'Aubigne and Postel score at 100 weeks	15 ± 3.2	14.9 ± 3.5	0.95	0.05
SF- 36 score (%) at 100 weeks	17.2 ± 19.1	17.6 ± 22.7	0.96	−0.04

**Table 2:** Comparison of the two groups

		No. of patients in group I	No. of patients in group II	*P* value
Gender	Male	3 (21.4)	8 (53.3)	0.07
	Female	11 (78.6)	7 (46.7)	
Severity of trauma	Mild	14 (100)	13 (86.7)	0.15
	Moderate	0	2 (13.3)	
Side involved	Right	7 (50)	7 (46.7)	0.85
	Left	7 (50)	8 (53.3)	
Garden's type	III	1 (7.1)	0	0.29
	IV	13 (92.9)	15 (100)	
Pauwels’ type	II	8 (57.1)	6 (40)	0.35
	III	6 (42.9)	9 (60)	
Singh's index	I	1 (7.1)	0	0.17
	II	10 (71.4)	8 (53.3)	
	III	2 (14.3)	7 (46.7)	
	IV	1 (7.1)	0	
Harris hip score at 100 weeks	Excellent	8 (57.14)	7 (46.6)	0.67
	Good	2 (14.28)	5 (33.3)	
	Fair	3 (21.42)	2 (13.3)	
	Poor	1 (7.14)	1 (6.6)	
d'Aubigne and Postel score at 100 weeks	Excellent	2 (14.28)	2 (13.3)	0.80
	Good	8 (57.14)	8 (53.3)	
	Fair	2 (14.28)	4 (26.6)	
	Poor	2 (14.28)	1 (6.6)	

Figures in parentheses are in percentage

Average Harris hip score at 6, 12 and 100 weeks was 64.7, 75.3 and 86 respectively in group I and 61.2, 74 and 85.5 respectively in group II. At 100 weeks group I showed good to excellent results (Harris hip score >80) in 71.4% (n=10), whereas group II showed good to excellent results in 80% (n=12) patients (P value = 0.67). However after exclusion of the patients with rheumatoid arthritis and osteoarthritis from group I, average Harris hip score at 6, 12 and 100 weeks was 67.9, 78 and 88.1 respectively and good to excellent results were observed in 91% (n=10) patients (P value = 0.42) at 100 weeks. Average d'Aubigne and postel score at 6, 12 and 100 weeks was 11.2, 13.9 and 15 respectively in group I and 11, 13.8 and 14.9 respectively in group II. At 100 weeks group I showed good to excellent results (d'Aubigne and postel score >14) in 71.4% (n = 10) patients whereas group II showed good to excellent results in 66.6% (n = 10) patients (P value = 0.80). However after exclusion of the patients with rheumatoid arthritis and osteoarthritis from group I, average d'Aubigne and postel score at 6, 12 and 100 weeks was 11.5, 14.3 and 15.5 respectively and good to excellent results were observed in 91 % (n=10) patients (P value = 0.32) at 100 weeks. Average SF-36 score at 6, 12 and 100 weeks was 41.4%, 26.7% and 17.2%, respectively in group I and 44.8%, 27.2% and 17.6%, respectively in group II (P value = 0.96). However after exclusion of the patients with rheumatoid arthritis and osteoarthrosis from group I, average SF-36 score at 6, 12 and 100 weeks was 40.2%, 24.42% and 14.6%, respectively in group I (P value = 0.72).

## DISCUSSION

Total hip arthroplasty addresses the inherent complications of nonunion and avascular necrosis and has been the recommended method of treatment in subcapital fractures of the neck of the femur in elderly patients.^27^,^28^ Early weight bearing can be permitted and complications secondary to recumbency can be avoided. Rehabilitation is fast but is at the cost of patient's own viable femoral head. Internal fixation preserves the patient's own viable femoral head. The disadvantages of the internal fixation include higher rates of avascular necrosis (10-17%) and nonunion (20-35%) requiring reoperation (20-36%).^9^,^10^

Present analysis has been carried out on 29 patients of displaced subcapital femoral neck fracture to evaluate the functional outcome of modified Pauwels’ intertrochanteric osteotomy and total hip arthroplasty in elderly patients. Although the study is small it is a step forward in preserving patient's own viable femoral head in femoral neck fracture in elderly patients. A small number and non-randomization of patients are the limitations of the present study where as a high incidence of union and a lower rate of avascular necrosis of femoral head after follow up of 2 years is the strength of the study.

The groups were comparable with respect to age, gender, side involved, severity of trauma, Singh's index, type of fracture and average time interval between injury and operation. A union rate of 93.3% (n = 14) of the fracture and 100% (n = 15) union of the osteotomy site have been achieved in an average of 15 and 14 weeks respectively in group II patients. In our previous study the authors have observed 94% and 100% union at the fracture and at the osteotomy site respectively.^29^ Other authors have also reported a union rate of 75% and 100% at the fracture site.^17^–^19^,^30^ Avascular necrosis of the femoral head was observed in 6.7% (n = 1) patients in the present series. The incidence of avascular necrosis has been reported between 8%-9.3% in various series of valgus intertrochanteric osteotomy with osteosynthesis.^17^–^19^ Intertrochanteric osteotomy is a biological procedure has previously been reported in various studies.^29^,^31^–^33^ Vascularity of the femoral head is not jeopardized further since procedure did not involve open reduction of fracture.

In the present study the average operation time for intertrochanteric osteotomy was significantly lower when compared with THR group (P value = 0.00001). Similarly requirement of blood transfusion in group I patients was significantly higher than patients of group II (P value = 0.001, significant). Excision of the femoral head, preparation and cementing of the acetabular and femoral components appeared technically demanding and time consuming. Recent studies have reported that excision of a 20-30 degrees intertrochanteric wedge of bone is sufficient to valgise and buttress the femoral head. Therefore, extensive preoperative planning is not required for intertrochanteric osteotomy and osteosynthesis of femoral neck fracture.^29^ Further almost half of the surgery can be done through the initial 2 inches long incision which minimizes blood loss. Authors are of the opinion that availability of the image intensifier has made the technique of intertrochanteric osteotomy simple. Thus total hip arthroplasty appeared to be more demanding procedure as compared to intertrochanteric osteotomy. Valgus osteotomy previously had been considered a very demanding surgical procedure, requiring anatomical reduction, precise calculation of the valgus angle and precise placement of the internal fixation device.^34^

Osteotomy can be done in conventional operation theatres whereas THR requires specialized operation theatre to reduce chances of infection. In the underdeveloped and developing countries only a fewer operation theatres suitable for THR are available. Internal fixation resulted in fewer postoperative complications, a shorter hospital stay and a reduced cost of treatment has been reported in other series also.^7^

Pauwels’ intertrochanteric osteotomy has been used to modify the mechanical environment about the proximal femur, thereby placing the head of femur at right angle to the resultant of body weight forces.^35^ It converts shearing forces at fracture site into compressive forces.^36^ No patient had coxa vara and a shortening of 2 cm in 6.7% (n = 1) has been well accepted by our patients. The valgus osteotomy has the advantages of correcting coxa vara and shortening simultaneously due to placement of proximal femur into a more valgus position and lateralization of distal osteotomy fragment. However, excessive valgus position of the head should be avoided to reduce stresses per unit area on the head of the femur. Lateralization of the distal osteotomy fragment increases the length of the abductor lever arm and overcomes the limp of the patient.^19^ Methews and Cabanela^37^ mention about postoperative limp, a complication that rarely has been reported in the literature, occurring in almost all of their patients. They related high rate of persistent limp to the mechanical changes of decreased offset and abductor lever arm caused by the valgus osteotomy and advice for avoiding excessive horizontalization.^37^

Retroversion of the femoral head caused limitation of extreme flexion in group II patients in the present series; an observation also reported in our previous studies.^29^,^32^ A malaligned acetabular cup and femoral head affects the chances of dislocation in patients of THR.^38^,^39^ A dislocation rate of 2-20% has been observed in various series of primary total hip replacement.^10^,^12^,^14^,^27^,^40^–^44^ Dislocation was observed in one of the patients in group I. Closed reduction failed to maintain the stability of the hip because of vertical inclination of the acetabular component. Revision surgery was performed in one of the patients in group II after failed osteosynthesis due to improper seating of the tip of blade in the head of femur. The authors feel that revision surgery in a patient of fracture of neck of femur with intertrochanteric osteotomy in situ is also a skilled procedure but revision of total hip arthroplasty usually is much more difficult and the results of revision THR are typically not as satisfactory as after a primary THR. Revision THR requires more operative time and more blood loss and the incidence of infection, thromboembolism, dislocation, nerve palsy and penetration and fracture of the femur are higher.^45^ Fair results in this patient after revision surgery substantiates our belief that femoral head preservation procedure should have a priority over THR in a patient with subcapital femoral neck fracture.

Squatting on toes and cross leg position is an essential component of day to day sociocultural activities of the people of third world countries. The external rotation deformity between 10°-20° in group II patients helped them squatting and sitting cross leg. Inability to squat on toes and cross leg position is another disadvantage of THR.

Collapse at the fracture site is a serious complication observed in one of our patients in group II. The tip of the blade penetrated the joint resulting in painful hip which necessitated THR. Conversion of failed osteosynthesis and intertrochanteric osteotomy into a THR does not compromise subsequent hip arthroplasty.^46^,^47^ Delayed rehabilitation is another disadvantage of intertrochanteric osteotomy as the full weight bearing is not permitted until radiological union of the fracture and osteotomy is achieved. However, functional outcome was comparable in both the groups at 100 weeks as per modified Harris hip score, d'Aubigne and postel score and SF-36 score. Raaymakers advocate intertrochanteric valgisation osteotomy, described by Pauwels as an excellent alternative for patients up to 65 years of age having nonunion of the femoral neck.^48^ Preservation of the femoral head in 93.3% (n = 14) patients with a lower incidence of avascular necrosis of 6.7% (n = 1) justifies the management of femoral neck fracture in elderly patients with modified Pauwels’ intertrochanteric osteotomy.

## CONCLUSION

Intertrochanteric osteotomy is a simple and biological procedure. 93.3% (n = 14) union can be achieved in femoral neck fracture in elderly patients and if the procedure fails it can be revised. Since the present non randomized series is small, a larger randomized series is suggested. Delayed rehabilitation is a disadvantage of intertrochanteric osteotomy and full weight bearing is not permitted until union of the fracture and osteotomy. External rotation deformity though preventable helps Indian patients sitting in squatting position in external rotation. Functional results of total hip replacement and intertrochanteric osteotomy are comparable and justify the usefulness of valgus intertrochanteric osteotomy with osteosynthesis in femoral neck fractures in elderly patients of sixty years and above.
